# Bearing Fault Diagnosis Method Based on Deep Convolutional Neural Network and Random Forest Ensemble Learning

**DOI:** 10.3390/s19051088

**Published:** 2019-03-03

**Authors:** Gaowei Xu, Min Liu, Zhuofu Jiang, Dirk Söffker, Weiming Shen

**Affiliations:** 1 School of Electronics and Information Engineering, Tongji University, Shanghai 201804, China; gaoweixu@tongji.edu.cn (G.X.); 1732919@tongji.edu.cn (Z.J.); 2 Dynamics and Control, University of Duisburg-Essen, 47057 Duisburg, Germany; soeffker@uni-due.de; 3 Key Laboratory of Embedded System and Service Computing, Tongji University, Shanghai 201804, China; wshen@ieee.org; 4 State Key Laboratory of Digital Manufacturing Equipment and Technology, Huazhong University of Science and Technology, Wuhan 430074, China

**Keywords:** bearing fault diagnosis, convolutional neural network (CNN), random forest (RF), continuous wavelet transform (CWT), ensemble learning

## Abstract

Recently, research on data-driven bearing fault diagnosis methods has attracted increasing attention due to the availability of massive condition monitoring data. However, most existing methods still have difficulties in learning representative features from the raw data. In addition, they assume that the feature distribution of training data in source domain is the same as that of testing data in target domain, which is invalid in many real-world bearing fault diagnosis problems. Since deep learning has the automatic feature extraction ability and ensemble learning can improve the accuracy and generalization performance of classifiers, this paper proposes a novel bearing fault diagnosis method based on deep convolutional neural network (CNN) and random forest (RF) ensemble learning. Firstly, time domain vibration signals are converted into two dimensional (2D) gray-scale images containing abundant fault information by continuous wavelet transform (CWT). Secondly, a CNN model based on LeNet-5 is built to automatically extract multi-level features that are sensitive to the detection of faults from the images. Finally, the multi-level features containing both local and global information are utilized to diagnose bearing faults by the ensemble of multiple RF classifiers. In particular, low-level features containing local characteristics and accurate details in the hidden layers are combined to improve the diagnostic performance. The effectiveness of the proposed method is validated by two sets of bearing data collected from reliance electric motor and rolling mill, respectively. The experimental results indicate that the proposed method achieves high accuracy in bearing fault diagnosis under complex operational conditions and is superior to traditional methods and standard deep learning methods.

## 1. Introduction

Nowadays, with the rapid development of modern industry, fault diagnosis technology, as a core of Prognostics and Health Management (PHM) system, is playing an increasingly important role in intelligent equipment maintenance [1,2]. Bearings are the essential components of most machinery and electrical equipment, their failures may result in considerable productivity and economic losses. Accurate and efficient bearing fault diagnosis can not only reduce the maintenance costs, but also improve the reliability and stability of equipment [2,3].

With the advent of the Internet of Things (IoT) and Cyber Physical System (CPS), a massive amount of historic data known as industrial Big Data is being collected from various equipment and systems. Therefore, research on data-driven fault diagnosis methods has attracted increasing attention [4,5,6]. Compared with signal-based and model-based fault diagnosis methods, they eliminate the complexity of signal processing and model establishment for different engineered systems [7].

Intelligent data-driven fault diagnosis methods usually consist of three critical steps: (1) data preprocessing (i.e., outliers elimination); (2) feature extraction and selection; (3) fault classification [8]. Feature extraction methods are commonly used to analyze waveform signal data including vibration data and extract signal-based features in the fault diagnosis of equipment [3,9,10,11]. However, there are still some redundant information in the extracted features. Then, feature selection techniques are adopted to significantly reduce feature dimensions, which can improve the classification efficiency while retaining important and representative features [12,13]. Finally, the selected features are used to diagnose faults by many fault classification methods based on traditional statistical and machine learning models [11,14,15]. It can be seen that traditional data-driven fault diagnosis methods have achieved some great progress. Nevertheless, there are still two important issues in these methods:

(1) The important and representative features containing enough fault information are manually extracted and selected from raw data, which depend heavily on prior knowledge and diagnostic expertise of signal processing techniques. In addition, the feature extraction and selection processes for different diagnostic problems are case sensitive, thus they are also time-consuming and laborious.
(2) The shallow architectures of traditional machine learning methods have problems in approximating nonlinear mapping relationship accurately in complex systems [7,16,17,18,19,20,21,22,23].

Deep learning is a new branch in the field of machine learning and can overcome the above-mentioned issues in fault diagnosis. It can replace the manual feature extraction and selection with automatic learning of representative features and construct input-output relationship in complex systems with a deep nonlinear network [16,17,18,19,20]. CNN model, as one of the most effective deep learning models, has also shown promising capability in useful feature learning and intelligent fault diagnosis [17,18,19,20,21,22,23]. It is widely accepted that only the extracted features in the last convolutional layer are most suitable as the input vector of the classifier in most researches and applications of CNN models [7,16,17,18,19,20,21,22,23]. Although the last layer contains more global and invariant high-level features for category-level fault classification, it is still questionable whether it is most appropriate to directly use high-level features for practical fault classification problems. The following four points need to be further considered:

(1) In many practical fault diagnosis applications, the training data and testing data are collected under different operational conditions, thus they are drawn from the different feature distribution [23]. It is well known that the extracted high-level features in CNN models are specific for particular dataset or task, while the low-level features in the hidden layers are universal and similar for different but related distribution datasets or tasks [24,25,26]. That is to say, low-level features extracted from training data in source domain are also applicable to test data in target domain. The generalization ability of the CNN-based fault diagnosis methods only taking high-level features into account would be poor. Therefore, low-level features in the hidden layers should be combined to obtain the better accuracy and generalization performance.
(2) Bearing health conditions with the same fault types under different damage sizes need to be classified in some practical applications. Since there are still some accurate details and local characteristics existing in low-level features which are not well preserved in high-level features [25,26], the classifier should make full use of multi-level features for accurate and complex fault diagnosis tasks.
(3) The extracted features in each layer are another abstract representation of raw data, thus the features in all layers can directly impact the diagnosis results.
(4) Sometimes, the extracted low-level features already contain enough fault information used for effective fault classification, there is no need to extract high-level features, which will cost more time and computer memory.

Recently, several studies have investigated the multi-level and multi-scale features aggregation of CNN models, which proves to be more effective in fault diagnosis and many other applications [24,25,27,28,29]. These models summarize multi-level or multi-scale features altogether into a category-level feature, then use it as the input of the full connection layer for more accurate classification. However, only a small proportion of low-level features in hidden layers are used in these models. In contrast, this paper takes the full advantage of the extracted multi-level features [30,31,32]. This is achieved by the following steps: firstly, A CWT-based signal-to-image conversion method is presented. The presented conversion method can effectively capture enough fault information under different conditions from nonlinear and non-stationary bearing vibration signals [33,34,35], and obtain the time-frequency spectrums of signals that can be regarded as gray-scale images. The problem of fault diagnosis can be solved by classifying these images. An improved CNN model based on LeNet-5 is then proposed to automatically extract representative multi-level features from these images. Finally, the extracted multi-level features at different layers in CNN are fed into multiple RF classifiers to classify faults independently, and the outputs of multiple classifiers are aggregated by the winner-take-all ensemble strategy to give the final diagnostic result.

The rest of this paper is organized as follows: Section 2 briefly reviews the related literature. Section 3 introduces the theory of CNN and RF. Section 4 presents the proposed method using CWT, CNN and RF. Section 5 analyzes the experimental results. Finally, the conclusions and further work are given in Section 6.

## 2. Literature Review

### 2.1. Data-Driven Fault Diagnosis

In the past few decades, many feature extraction methods in the time domain, frequency domain, and time-frequency domain were applied to extract fault features of the signals and assist the fault classification methods to diagnose faults. Among them, time-frequency domain methods are most effective for analyzing non-stationary signals like bearing vibration signals. Lei et al. [9] used the empirical mode decomposition (EMD) method to extract features from the bearing vibration signals and presented a kurtosis-based method to select the sensitive features for fault diagnosis. Lin et al. [10] used an improved EMD method to extract features for nonlinear, non-stationary and composite signals. He et al. [18] employed a short-time Fourier transform (STFT) method to preprocess the acoustic emission signals obtained from bearing tests. Feature selection methods such as linear discriminant analysis (LDA) and principal component analysis (PCA) were widely utilized to select the most representative features from the extracted features. Sun et al. [12] presented a fault diagnosis method based on decision tree and PCA, PCA is applied to reduce features after feature extraction. Jin [13] proposed a trace ratio LDA-based motor bearing fault diagnosis method, where high-dimensional fault data is dealt with using LDA for dimension reduction. Traditional data-driven fault diagnosis methods based on machine learning techniques have achieved significant success, in which support vector machine (SVM), k-nearest neighbor (KNN) and artificial neural network (ANN) are the most widely applied. Yang et al. [14] used the SVM to identify the fault patterns of the roller bearings classifiers with the fault characteristic vectors. Ngaopitakkul et al. [11] constructed a decision algorithm based on ANN for fault diagnosis on single-circuit transmission line. Pandya et al. [15] proposed a fault diagnosis technique based on an efficient KNN classifier using asymmetric proximity function. Flores-Fuentes [36] et al. performed an evaluation of different artificial intelligence methods for a machine vision application.

### 2.2. Deep Learning and CNN

Deep learning is an advanced technology that can automatically learn essential and representative features of raw data. Feature learning can be supervised, semi-supervised or unsupervised [16]. Recently, some deep learning models including deep belief network (DBN), deep auto encoder (DAE) and CNN have been used in the research domain of fault diagnosis [17,18,19,20,21,22]. Shao et al. [17] proposed an optimization DBN for bearing fault diagnosis, and the model is applied to analyze the experimental signal of a rolling bearing. Qi et al. [19] presented a DAE-based fault diagnosis method of rotating machinery, which can effectively mine abstract and sensitive high-level features. CNN is a typical representative of deep learning models, which has also achieved a considerable performance in fault diagnosis tasks. Wen et al. [7] presented a new CNN model based on LeNet-5 for fault diagnosis, a novel data preprocessing method without any predefined parameters is adopted to extract the features of raw signals. Xia et al. [21] proposed a CNN-based method for fault diagnosis of rotating machinery to achieve higher accuracy. Lee et al. [22] presented a CNN model using a receptive field to extract fault features, which has been successfully applied to fault diagnosis.

In the past few years, some researchers have made significant efforts on making full use of features in the multi layers of CNN and have achieved considerable progress. For example, Ding et al. [25] combined the features in the max-pooling layer and the last convolutional layer to obtain more robust multi-scale features with precise details. Sun et al. [27] combined features in the top layers with some hidden neurons to increase the generalization capacity of the deep learning model. Lee et al. [28] proposed a CNN-based architecture for music auto-tagging that consists of multi-level and multi-scale features, the proposed architecture outperforms the current methods and is also useful in transfer learning. It can be found that sometimes the CNN models with multi-level or multi-scale features tend to be more precise compared to the CNN model with only the high-level feature. 

### 2.3. Ensemble Learning

Ensemble learning, also called multi-classifier system and committee-based learning, uses multiple base classifiers to obtain better classification accuracy and generalization performance. To be specific, a set of diverse individual classifiers are generated. In order to improve the quality and robustness of the individual classifiers, classification results of the individual classifiers are aggregated by predefined strategies. There are several widely-used ensemble strategies, such as majority voting, Bayes optimal classifier, Bootstrap aggregating (bagging), Boosting, Stacking, winner-take-all and other user-defined strategies [37,38,39]. RF is a typical ensemble classifier that is constructed by a multitude of decision trees. RF classifier has been attracting increasing attention due to its excellent classification accuracy and high efficiency. For example, Santur et al. [40] presented a RF based diagnosis approach for Rail fault inspection due to high accuracy and rapid functioning of RF method. A number of studies have recently proven the superiority of ensemble learning technique for fault classification tasks. Zhang et al. [31] presented a fault diagnosis method based on fuzzy entropy and ensemble SVM, fuzzy entropy is employed to extract the hidden features and the ensemble SVMs is constructed to classify faults. Shao et al. [32] presented an ensemble DAE model for bearing fault diagnosis, a combination ensemble strategy is applied to provide more precise and stable diagnosis results.

## 3. Theoretical Background

### 3.1. CNN

In general, CNN is always composed of multiple convolution layers, pooling layers and fully connected layers, an input layer, and an output layer. The input layer contains the data or images to be processed. At a convolutional layer, a set of new feature maps are obtained. For each feature map, firstly, the input is convolved with a kernel which has a local receptive field. Then, a bias term is added to the convolution result. Finally, an activation function is applied. The operation is defined as:

(1) $$x_{i}^{j}=f\left(\sum_{k=1}^{n}W_{i,k}^{j}\times x_{k}^{j-1}+b_{i}^{j}\right),$$

where $x_{i}^{j}$ donates the i-th output feature map of j-th level; $x_{k}^{j-1}$ donates the k-th input feature map of the (j−1)-th level; $W_{i,k}^{j}$ is the convolution kernel between the i-th output feature map at the j-th layer and k-th input feature map at the (j−1)-th layer; n is the number of the input feature maps; $b_{i}^{j}$ is the bias of ith output feature map at the j-th layer; f(·) is the activation function. The most commonly-used functions are tangent, ReLU, and sigmoid function [12]. The ReLU function can increase the nonlinearity of CNN. It is adopted in this paper due to its excellent performance when applied in CNN. ReLU is defined as:

(2) $$x_{i}^{j}=max\left(0,x_{i}^{j^{\prime }}\right),$$

To decrease the number of parameters in CNN, the convolution kernels for the same feature map share the same weight vectors and bias. Generally, a max-pooling layer is added to each convolutional layer, which produces lower-resolution feature maps by sub-sampling operation. Max-pooling is defined as:

(3) $$x_{i}^{a,b}=max(x_{i}^{a^{\prime },b^{\prime }}:a\leq a^{\prime }<a+p,b\leq b^{\prime }<b+p),$$

where $x_{i}^{a^{\prime },b^{\prime }}$, $x_{i}^{a,b}$ are the (a,b) pixel in the i-th feature map before and after max-pooling operation, respectively; Further p is the stride size of pooling window and p should be larger than 1. Note that excessive value of p may result in a certain degree of information loss. The pooling layer decreases the size of the input feature maps while maintaining the number of feature maps. With the sub-sampling technique, the number of parameters in CNN is further reduced. 

The last pooling layer is followed by a fully-connected layer. Each neuron in the fully-connected layer is connected to all feature maps in the last pooling layer. The high-level feature in the fully-connected layer are extracted and are taken as the input of the output layer. Then, the prediction output of the CNN model is generated. Finally, the parameters {W,b} (weight vectors and bias) of the network are fine-tuned by minimizing the loss function, with which the error between the predicted output y and the targeted output t can be calculated as

(4) $${\left\{W,b\right\}}^{*}=arg{min}_{\left\{W,b\right\}}\frac{1}{n}\sum_{i=1}^{n}J\left(t,y\right),$$

where ${\left\{W,b\right\}}^{*}$ is the optimized parameters; n is the number of the labelled samples; and J is a loss function. In addition, the gradient-based supervised training of the network is performed through the back propagation algorithm [41].

### 3.2. Ensemble Learning and RF

**Figure 1:**
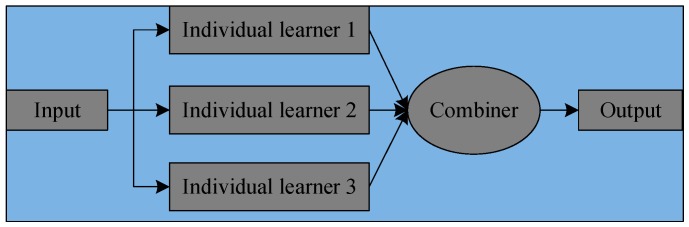
The architecture of ensemble learning.

The architecture of ensemble learning is shown in Figure 1. A number of individual learners are generated by training input data with different learning algorithms, which are decision tree, SVM, RF and so on. In addition, the learners can be generated in a sequential style and a parallel style, two representative ensemble learning methods are boosting and bagging. Then the results of individual learners are aggregated by specific combination strategies. 

Decision tree is a simple and intuitive method for classification and regression tasks. A decision tree is a flowchart-like structure with a root node, internal nodes and leaf nodes. The root node and internal nodes create binary splits and each leaf node is connected to the root node through the internal nodes. For an exhaustive study of decision trees the reader is suggested to consult other references like [42].

RF is one of the most popular ensemble learning methods, which consists of a bootstrap aggregating (bagging) of N decision trees with a randomized selection of features at each split. Given a training dataset, the RF algorithm is as follows:

*1. For* n=1 to N:

*(a) Generate a bootstrap sample with replacement from the training dataset.*

*(b) Build a random-forest tree based on each bootstrap sample data, randomly select* M *predictors at each internal node and pick the best split using only the* M *predictors rather than all predictors.*

*2. Predict new data by the averaging of the* N *trained trees for regression tasks, or the majority vote of the* N *trained trees for classification tasks.*

The variance of the RF model is reduced without adding the bias due to the bootstrapping procedure. Therefore, while the prediction accuracy of the decision tree is easily affected by the random noise in data, the RF model is not sensitive to the noise and has better performance in term of accuracy. On the other hand, the subset of predictors is considered, so RF is easier to tune parameters than to use all predictors. 

## 4. Proposed Method

**Figure 2:**
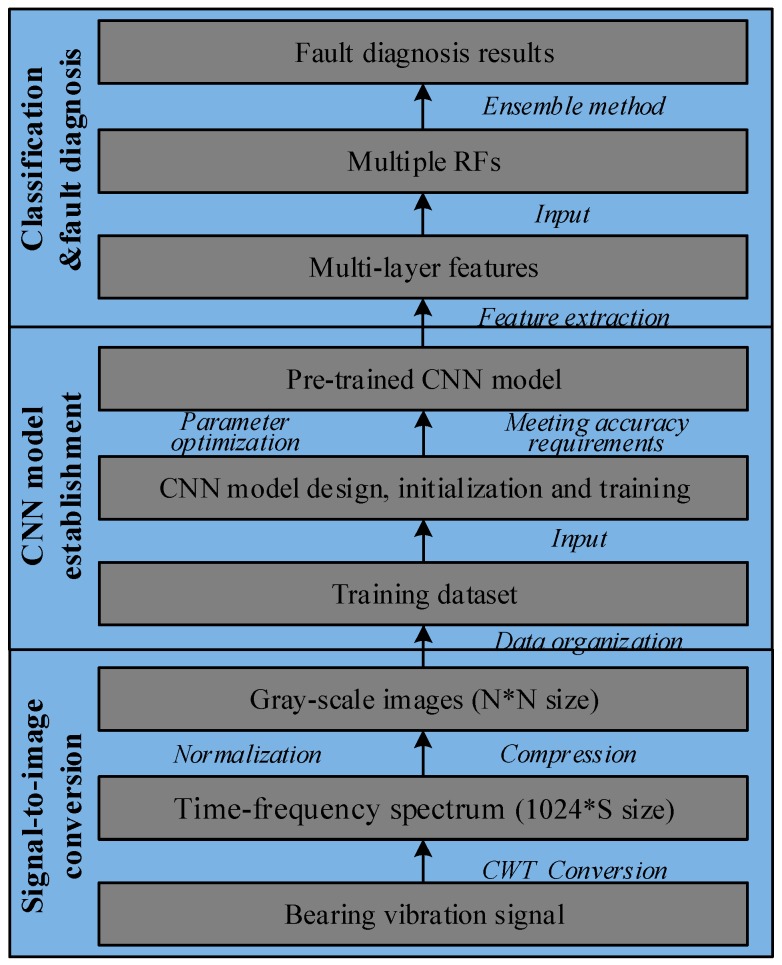
The flowchart of the proposed method.

In this section, a novel data-driven rolling bearing fault diagnosis method based on CWT, CNN and RF is presented. The flowchart of this method is shown in Figure 2. 

It contains three major steps. Firstly, the raw vibration signal data from bearing dataset are converted into time-frequency spectrums using CWT, which are presented in the form of gray-scale images. The converted images contain enough fault information that is beneficial for fault classification. Then the images are compressed in order to reduce the computational complexity. Secondly, an improved CNN model based on LeNet-5 [43] is designed; the parameters of the CNN model are randomly initialized; and the CNN model is pre-trained on the converted images. The pre-trained CNN model is used as the feature extractor to learn the representative multi-level features from these images. Next, the extracted multi-layer features in different layers are fed into multiple RF classifiers separately and the results of multiple RF classifiers are combined by the winner-take-all ensemble method. Finally, the combined classification results are used as the final diagnostic result.

### 4.1. Signal-to-Image Conversion Based on CWT

A wavelet is a normalized function $\psi \left(t\right)\in L^{2}\left(R\right)$ of finite energy and zero average:

$$\bar{\psi }\left(t\right)=\int_{-\infty }^{+\infty }\psi \left(t\right)dt=0$$

(5) $$||\psi (t){\left|\right|}^{2}=\int_{-\infty }^{+\infty }\left|\psi \left(t\right)\right|dt=1,$$

The basic wavelet function ψ(t) is usually called the mother wavelet function, based on which, a family of time-scale wavelets $\psi_{a,b}\left(t\right)$ can be formulated by scale and translation adjustment described by:

(6) $$\psi_{a,b}\left(t\right)={\left|a\right|}^{-\frac{1}{2}}\psi \left(\frac{t-b}{a}\right)\;\;\;\;a,b\in R,\;a>0,$$

where a and b represent the scale and translation factors, respectively. Specifically, the scale factor a either stretches or compresses the wavelet function to change the oscillating frequency and the translation factor b changes the position of time window. The longer scale stretches the wavelet and decreases the frequency, and the smaller scale compresses the wavelet and increases the frequency. 

For an arbitrary signal function $f\left(t\right)\in L^{2}\left(R\right)$, the corresponding CWT is defined as:

(7) $$CWT_{f}\left(a,b\right)=\langle f\left(t\right),\psi_{a,b}\left(t\right)\rangle ={\left|a\right|}^{-\frac{1}{2}}\int_{-\infty }^{+\infty }f\left(t\right)\bar{\psi }\left(\frac{t-b}{a}\right)dt,$$

where $\bar{\psi }\left(t\right)$ is the complex conjugate of the mother wavelet function ψ(t), $CWT_{f}\left(a,b\right)$ is the inner product of f(t) and $\psi_{a,b}\left(t\right)$, which reflects the similarity between the signal function and wavelet function. Wavelet functions have focal features and time-frequency localization properties and can effectively capture non-stationary signal characteristics. There are many mother wavelet functions, such as Haar, Meyer, Coiflet, Symlet, Gabor, and Morlet. Among them, Morlet wavelet has been proven to be superior to others in term of non-stationary rolling bearing vibration signal analysis due to its similarity to the transient impulse components of bearing faults [44,45,46,47]. Thus it is chosen as the mother wavelet function for bearing fault diagnosis in this paper. The Morlet wavelet in time domain is defined as:

(8) $$\psi \left(t\right)=exp\left(-\beta^{2}t^{2}/2\right)\cos\left(\pi t\right),$$

where β is the only parameter which controls the shape of the basic wavelet. As the β value increases, the resolution of time domain increases and the resolution of frequency domain decreases. CWT using Morlet can fully capture the signal characteristics and obtain good resolution in both time and frequency domains [45]. In this paper, continuous Morlet wavelet transform converts the one-dimension vibration signals in time-domain into two-dimension time-frequency spectrum with abundant condition information. 

**Figure 3:**
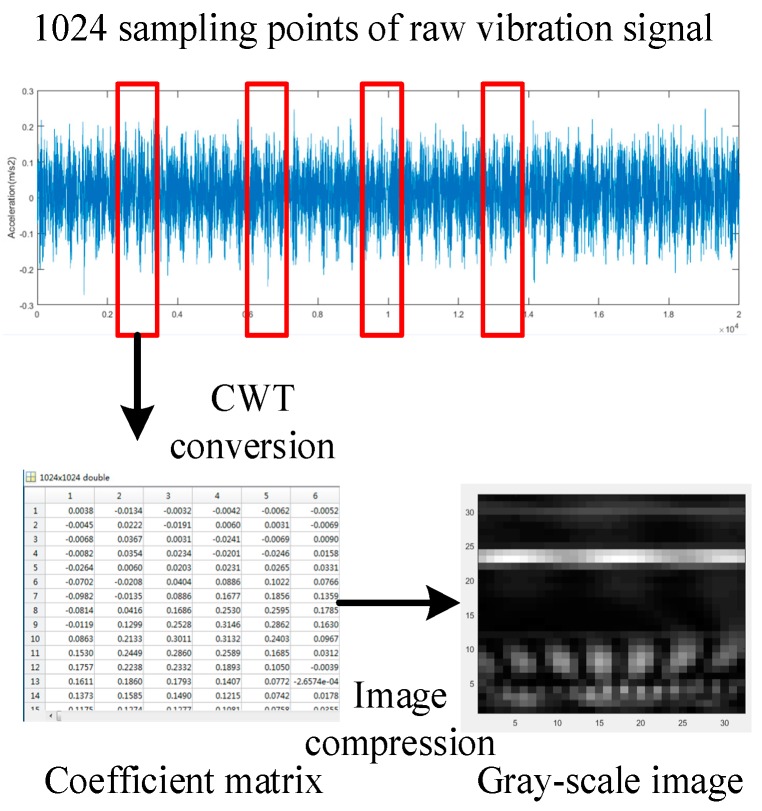
The specific process of signal-to-image conversion method.

The specific process of signal-to-image conversion method is shown in Figure 3. Firstly, 1024 continuous points are sampled randomly from the raw signals [4,7,21,25]. Then the 1024 points are converted into a 1024 × S time-frequency spectrum that is consisted of coefficient matrices by the continuous Morlet wavelet transform. Here S indicates that the value of the scale factor a range from 1 to S. In a practical application, as long as the value of S is sufficiently large, sufficient raw signal characteristics can be obtained. Finally, the time-frequency spectrum is present in the form of gray-scale image. However, the CNN model usually has difficulty in dealing with 1024 × S image, and the extra-large size of the image may result in considerable computational complexity. A simple image compression method based on bicubic interpolation [48] is used to decrease the size of the image. In this paper, the size of the compressed gray-scale image varies due to the different volumes of the signal data.

### 4.2. Design of the Proposed CNN

Based on the gray-scale images converted from raw vibration signals, a CNN model based on LeNet-5 is designed and pre-trained for feature learning. The training dataset is used to update the parameters of CNN by back-propagating errors. Once the training process finishes, the representative multi-level features can be extracted automatically from these images.

**Figure 4:**
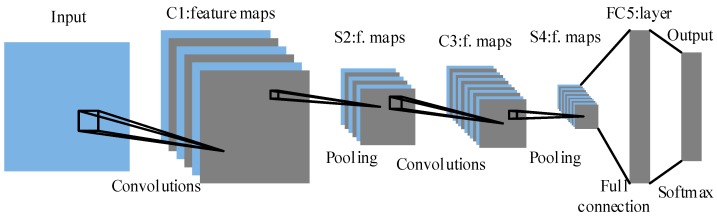
Architecture of the proposed CNN.

Figure 4 illustrates the architecture of the proposed CNN which contains seven layers, including one input layer, two convolutional layers, two pooling layers, one fully-connected layer, and one softmax output layer. 

The detailed structure of each layer can be altered to improve the classification accuracy for different cases. On the basis of the analysis in Section 3.1, the size of input image should be as small as possible while guaranteeing that the image contains enough fault information. Through the convolution between the $n_{1}$ kernels of size $c_{1}\times c_{1}$ and the input image, $n_{1}$ feature maps of size $\left(n_{1}-c_{1}+1)\times (n_{1}-c_{1}+1\right)$ in layer C1 are obtained with the ReLU activation function. Each feature map in C1 is subsampled to the corresponding feature map in layer S2 by the max-pooling operation of size $s_{1}\times s_{1}$. Layer S2 is a pooling layer which is composed of the same number of feature maps of size $\left((n_{1}-c_{1}+1\right)/s_{1})\times ((n_{1}-c_{1}+1)/s_{1})$. Layer C3 and S4 are formed in a similar way. Layer FC5 is a fully-connected layer and the size of each feature map in this layer is 1 × 1. Each pixel in layer FC5 is connected to a variable sized neighborhood in all feature maps in S4. Finally, a softmax output layer is followed to output the classification results. 

Especially, the feature maps $x^{\left(i\right)}$ in layer FC5 are put into the softmax classifier and the probability distribution of the input sample belonging to each class is calculated as:

$$p(y^{\left(i\right)}=j|x^{\left(i\right)};\theta )=\frac{exp\left(\theta_{j}^{T}x^{\left(i\right)}\right)}{\sum_{l=1}^{k}exp\left(\theta_{l}^{T}x^{\left(i\right)}\right)}$$

(9) $$y=argmax_{j}\;p(y^{\left(i\right)}=j|x^{\left(i\right)};\theta ),$$

where i=1,2,…,n; n is the number of training data; j=1,2,…,k; *k* is the dimension of output layer and it should be set as the number of fault types. Additionally, θ express the parameters of the softmax classifier. The loss function of the softmax classifier is defined as:

(10) $$J\left(\theta \right)=-\frac{1}{n}\left[\sum_{i=1}^{n}\sum_{j=1}^{k}I\left\{y^{\left(i\right)}=j\right\}\right]logp\left(y^{\left(i\right)}=j|x^{\left(i\right)};\theta \right),$$

where I{.} is the indicator function. The parameters of the proposed CNN are optimized by solving for the minimum of J(θ) using the mini-batch stochastic gradient descent method.

### 4.3. Ensemble Classification

**Figure 5:**
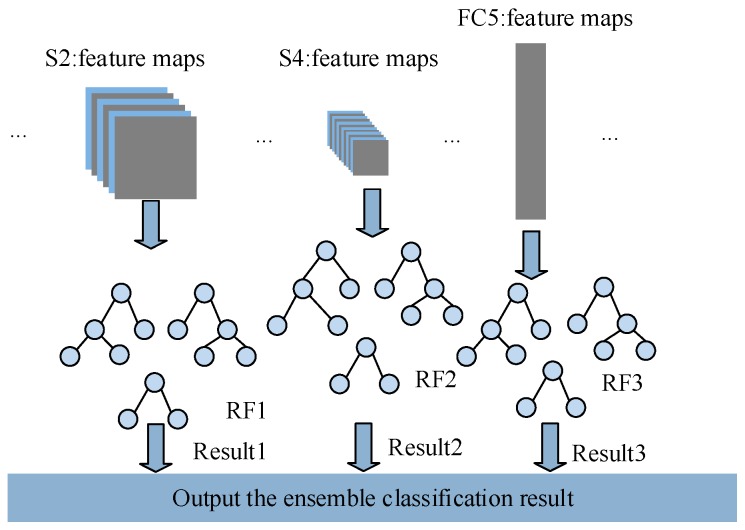
The ensemble of multiple classifiers.

It has been well stated in the introduction that the full use of the multi-level features can improve the accuracy and generalization performance. Therefore, multi-level features derived from the pre-trained CNN are taken into consideration in this paper. As shown in Figure 5, the extracted features of layer S2, S4 and FC5 are fed to three RFs separately, and each RF classifier consists of 50 decision trees in this paper. Here the trained CNN is worked as the trainable feature extractor and the RF is used as the base classifier.

Each RF classifier is trained independently using the feature maps in different layers of CNN. Once all the RF classifiers are completely trained, the outputs of three RF classifiers can be combined to obtain better classification performance by the winner-take-all ensemble strategy [38]. This is because ensemble learning technique has a great impact on the improvement of model performance and has been widely applied to fault diagnosis [30,31,32]. In the winner-take-all ensemble strategy, several base RF classifiers are competing with each other, so the ensemble output is consistent with the output of the base RF classifier which obtains the best classification performance in different layers. The general procedure of the proposed fault diagnosis method based on CNN and RF ensemble is given in Algorithm 1.

**Algorithm 1** General Procedure of the Proposed Method**Input:** Given the bearing dataset composed of vibration signal samples, the architecture and hyper-parameters of the proposed CNN and the RF classifier **Output:** The diagnostic results, and the testing accuracy and efficiency **Step 1: Generate the training dataset and the test dataset** 1.1: Obtain the spectrums of n vibration signal samples for training and m samples for testing using the signal-to-image conversion method. 1.2: Use the spectrums to generate the training dataset $X_{s}$ and the test dataset $X_{t}$. **Step 2: Construct and train the CNN for multi-level features extraction** 2.1 Construct the CNN and initialize the parameters θ{W,b} (weight vectors and bias) of the CNN randomly. 2.2 Train the CNN on the training dataset $X_{s}$, calculate the outputs of layers in the CNN using Equations (1)–(4). 2.3 Calculate the outputs of the output layer and the loss function of softmax classifier using Equations (9) and (10). 2.4 Optimize the parameters θ{W,b} through minimizing the Equation (10) using the mini-batch stochastic gradient descent method. 2.5 Repeat (2.2)–(2.4) until meeting the training requirements, and finish the training process of the CNN. 2.6 Extract the features $x^{2}$, $x^{4}$, $x^{5}$ in layer S2, S4 and FC5 from the trained CNN, and $x^{j}$ donates the output feature map of jth level. **Step 3: Use multi-level features to train multiple RF classifiers** 3.1 Use the extracted features $x^{2}$, $x^{4}$, $x^{5}$ to train the classifiers RF1, RF2, RF3, respectively. 3.2 Output the diagnostic results of three RF classifiers separately. **Step 4: Output the final result using the ensemble method** Aggregate the outputs of three RF classifiers with the winner-take-all ensemble strategy to output the final diagnostic result. **Step 5: Validate the performance of the proposed method** Validate the performance of the proposed method on test dataset $X_{t}$ and output the testing accuracy and efficiency of the proposed method

## 5. Experimental Results

In order to evaluate the effectiveness of the proposed method for bearing fault diagnosis, two case studies are conducted with two bearing datasets from reliance electric motor and rolling mill, respectively. All the experiments are carried out with Matlab R2018a on a desktop computer equipped with an Intel 4-core 2.3 GHz processor, 8 GB memory and a 500 GB hard disk.

### 5.1. Case Study 1: Bearing Fault Diagnosis for Reliance Electric Motor

#### 5.1.1. Experimental Setup and Dataset

In this case study, the effectiveness of the proposed method is validated by the public bearing dataset provided by the CWRU Bearing Data Center [49]. The vibration signal data in this dataset are collected from a 2-hp reliance electric motor, the accelerators are installed at the drive end, the fan end, and the base, respectively. In this paper, only the signal data at the drive end are taken and analyzed. The vibration data are sampled at the frequency of 12 kHz under different loads, ranging from 0 to 3. There are three fault types of drive end bearing, which are inner race fault (IF), ball fault (BF), and outer race fault (OF). In addition, there are three different damage sizes (0.18, 0.36 and 0.54 mm) for each fault type. Therefore, ten health conditions including one normal condition (NO) and nine fault conditions are included in the dataset.

In this experiment, two datasets are generated. Firstly, for each health condition, 50 samples with 1024 data points are randomly selected under each load condition in the training dataset. That is to say, there are 2000 training samples with 10 health conditions under four load conditions. On the other hand, in the test dataset, 2000 samples are randomly selected in the same manner. In addition, another dataset is generated to further verify the robustness and generalization ability of the proposed method. Furthermore, the training and testing samples of Dataset II are selected under different operational loads. 1500 samples with 10 health conditions are randomly selected under the loads of 0, 1, and 2 in the training dataset, while the test dataset is composed of 500 samples with 10 health conditions under the load of 3. More details of the two datasets, named Dataset I and Dataset II, are listed in Table 1. It should be noted that it is important to randomize the order of all the samples in the dataset before training.

The raw vibration signal data are converted to gray-scale images by CWT and the scale factor of CWT is set as 1024. Because of the volume of the signal data in this case study, all the images are compressed to the size of 32 × 32 by the imresize function based on bicubic interpolation in Matlab. In this case study, the detailed structure of the CNN model is shown in Table 2. The performance of CNN model reaches the peak with the above configuration. Here C1(6@28 × 28) denotes that 6 feature maps of size 28 × 28 in layer C1 are used, C1(6@5 × 5) denotes that the layer C1 is obtained by the convolution between the 6 kernels of size 5×5 and the input layer. In addition, S2(2 × 2) denotes that the pooling layer S2 is obtained by the max-pooling operation on layer C1 of size 2 × 2. The parameters of the CNN model are optimized in a heuristic way [22]. The value of initial learning rate is selected from 0.0001 to 0.1 with the step of 0.005. As a result, the model achieves the best training performance when the initial learning rate is set as 0.05. Considering the number of samples in training dataset, the batch sizes are selected among these values: 30, 60, 90 and 120, the model performance is checked for each value. The best batch size is 120 for dataset I, 90 for dataset II. The number of epochs is set as 60.

#### 5.1.2. Results and Discussion

**Figure 6:**
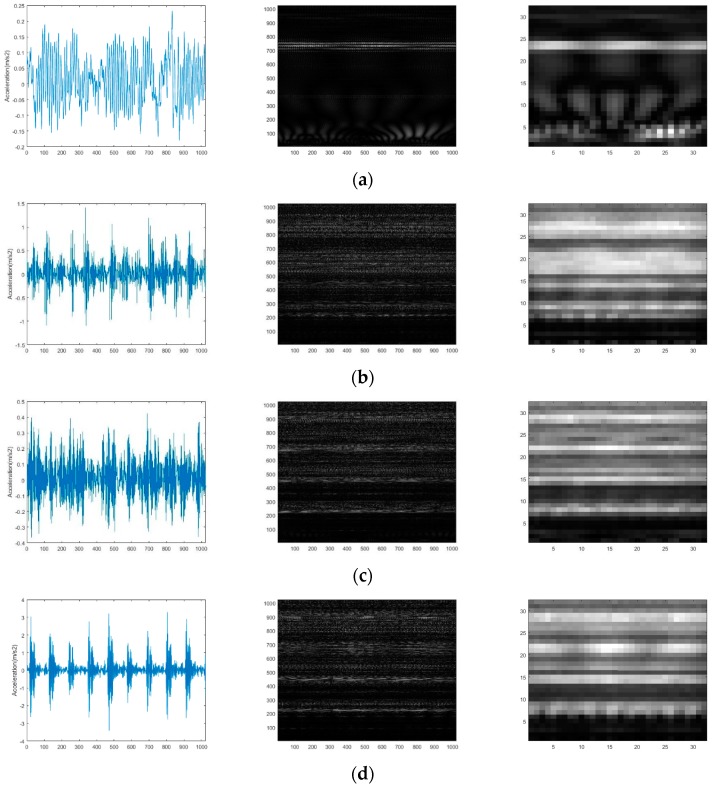
The raw vibration signal waveform and conversion results: (**a**) normal condition; (**b**) inner race fault condition; (**c**) ball fault condition; (**d**) outer race fault condition.

The raw vibration signal waveform, conversion results before and after compression of one normal condition and three representative fault conditions (damage size = 0.18 mm, load = 0) are shown in Figure 6. It can be found that it is difficult to distinguish the health conditions according to the vibration signal waveform in the time domain. However, an obvious difference between the gray-scale images of different health conditions no matter before and after compression can be detected. Therefore, the problem of fault diagnosis can be solved by classifying these images.

**Figure 7:**
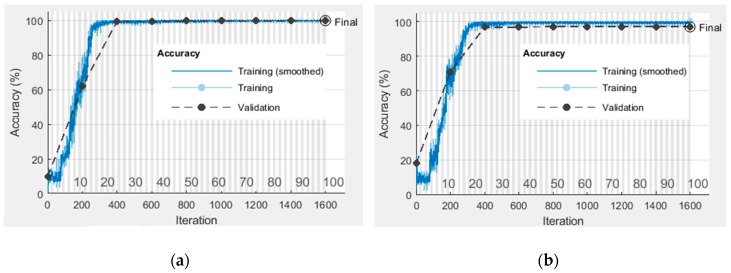
The training accuracy curves of the proposed CNN model: (**a**) Dataset I; (**b**) Dataset II.

The weights and bias of the CNN model are optimized through training the gray-scale image samples. The training accuracy curves for dataset I and II are shown in Figure 7. In can be seen that the proposed CNN model can converge with a limited number of iterations so that the training accuracy for two datasets both can reach almost 100%.

**Figure 8:**
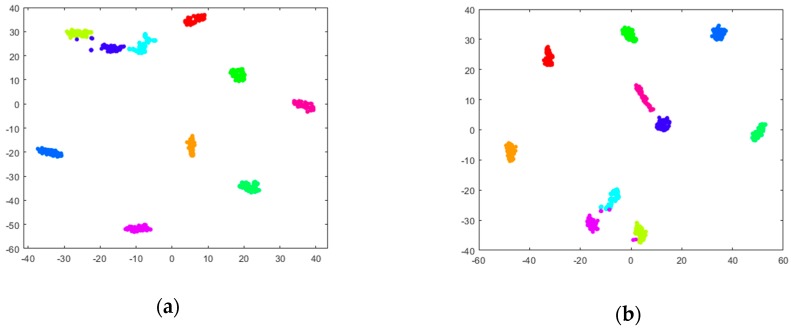

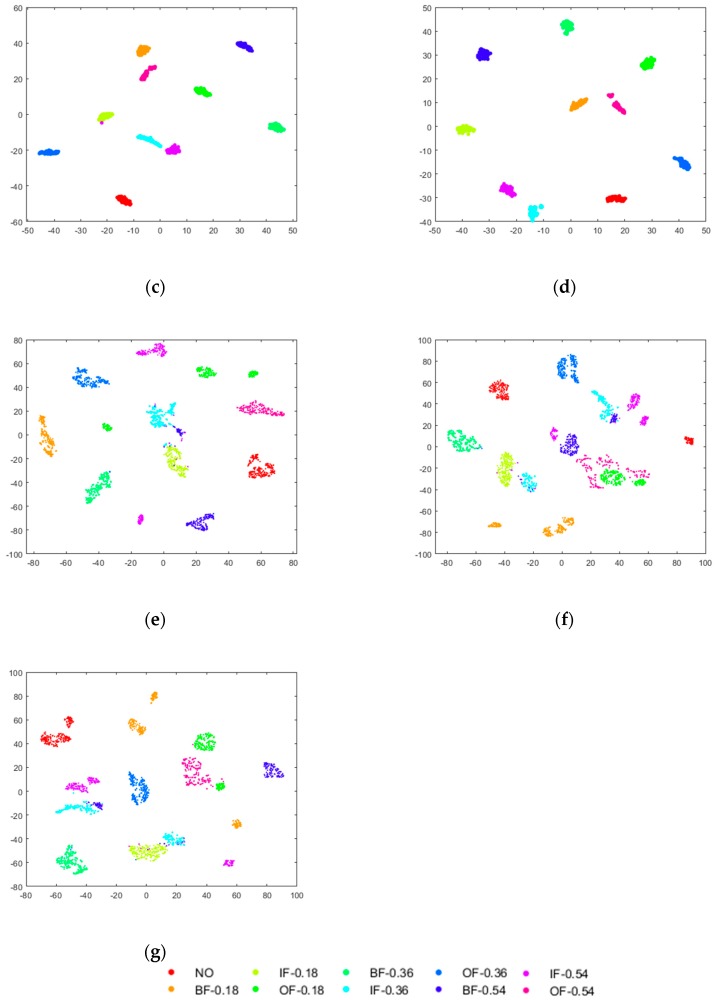
Visualization of multi-level features via t-SNE: (**a**) FC5 (Load: 0); (**b**) FC5 (Load: 1); (**c**) FC5 (Load: 2); (**d**) FC5 (Load: 3); (**e**) FC5 (Loads: 0–3); (**f**) S2 (Loads: 0–3); (**g**) S4 (Loads: 0–3).

To demonstrate the representative and robust feature extraction ability of the proposed CNN model, taking the dataset I for example, the dimension of the extracted features is reduced to two for visualization by the t-distributed stochastic neighbor embedding (t-SNE) method. The t-SNE technique [50], an efficient nonlinear dimensionality reduction method, is used for embedding high-dimensional data for visualization in a low-dimensional space. The two-dimensional visualizations of the extracted features in the last layer under load 0, 1, 2, 3, and loads 0–3, also the multi-level features under loads 0–3 are shown in Figure 8, in which different colors represent different health conditions. As shown in Figure 8a–e, it can be found that under all different operational conditions the extracted high-level features for the same health conditions are relatively centralized except very little of samples, while the features for the different health conditions are separated. Therefore, it can be concluded that the proposed CNN model has strong ability in extracting the representative features, and is certainly effective for multi-classification problems in fault diagnosis. On the other hand, by the contrast analysis between Figure 8e–g, it is worth mentioning that the vast majority of samples belonging to the same health conditions can also be well gathered together in the hidden layers. In addition, the two-dimensional visualizations of the extracted features in different levels under the complex operational condition (loads:0–3) are different from each other, thus concluding that the low-level features are also useful for fault classification and the multi-level features can contribute different knowledge to the diagnosis results.

**Figure 9:**
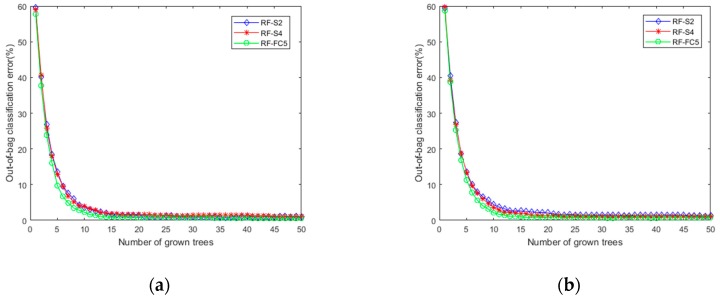
The training error curves of 3 RF classifiers: (**a**) Dataset I; (**b**) Dataset II.

The extracted feature maps in layer S2, S4 and FC5 are fed into three RF classifiers separately and the training error curves are shown in Figure 9. It should be noted that the training errors for the three RF classifiers are close to zero, which further indicates that the feature maps in the hidden layers also contains important information that can contribute to the diagnosis results.

Based on the learned multi-layer features, the diagnosis experiments on both datasets are executed 10 times. The mean and standard deviation of the diagnostic accuracy of softmax, RF1, RF2, RF3 and ensemble classifier are shown in Table 3. As shown in Table 3, it can be concluded that the method using different classifiers in different layers achieves remarkable results. The method using RF2 classifier in layer S4 has the highest accuracy at 99.73% for Dataset I and the method using RF2 classifier in layer S4 has the highest accuracy at 99.08% for Dataset II. Especially for Dataset II, the accuracies of the classifiers using the feature maps in other layers are range from 95.02% to 98.26%, which are inferior to those of the ensemble classifier. It is worth emphasizing that the training and testing samples of Dataset II are selected under different loads, that is to say, feature distribution of training data is different from that of testing data, so the clustering performance of different layers does not have direct impact on classification accuracy for Dataset II. From the results, the different loads in training and test dataset cannot affect the classification accuracies of the proposed method, and the proposed method promotes the diagnostic accuracy a lot compared to the standard CNN model for Dataset II. This is due to local characteristics and accurate details of faults exist in low-level features which are not well preserved in high-level features. Therefore, it is not always the best way to directly use the high-level features for bearing fault diagnosis, where health conditions with different damage sizes from the same fault type need to be classified. On the other hand, the extracted low-level features in the hidden layers are universal and similar for different but related distribution training and test datasets, while the more abstracted high-level features are sensitive and specific for particular fault diagnosis dataset or task. Furthermore, the standard deviation of the proposed method is 0.109% for dataset I, 0.379% for Dataset II. It can be concluded that the introduction of the ensemble method of multiple RF classifiers using multi-level features can improve the robustness and generalization ability of the proposed method. 

#### 5.1.3. Comparison with Other Methods

Five fault diagnosis methods based on traditional machine learning models, including BPNN (back-propagation neural network), SVM, and standard deep learning models including DBN, DAE, CNN are also implemented in this case study for comparison purpose. Manual feature extraction process is conducted in fault diagnosis methods based on BPNN, SVM. Four time-domain features and six frequency-domain features are manually selected and assist BPNN and SVM to diagnose faults. For more details of the adopted manual feature extraction technique, please refer to Ref. [9]. The representative features are automatically extracted and selected in fault diagnosis methods based on standard DBN [17], DAE [20] and CNN with the aid of the corresponding data pre-processing methods.

All the comparison experiments are conducted 10 times on the dataset I and II. In order to verify the superiority of the proposed method, the mean accuracy, average computation time of the above-mentioned methods are compared to that of the proposed method. The comparison results are shown in Table 4.

It can be seen that the mean diagnosis accuracies of the traditional machine learning-based methods are significantly worse than those of the proposed method for the two datasets, which results from the shallow architecture of the traditional machine learning-based methods not being able to explore the complex relationships between the health conditions and the signal data. In addition, the diagnosis performance of these methods depends heavily on manual feature extraction. However, the features extracted manually show a poor ability of representing raw data. The proposed method also achieves better results compared to the other deep learning based methods, especially for Dataset II, which further demonstrates the superiority of the proposed method. On the other hand, although the average computation time of the proposed method is much longer than that of traditional machine learning-based methods, but the average time spent on signal preprocessing and manual feature extraction in these methods is not taken into account, which is a very time-consuming and labor-intensive task. Additionally, compared with traditional deep learning based methods, the proposed method need more computation time because ensemble requires more computation. However, with the development of the hardware technology, a bit more computation time less than 10 seconds can be ignored due to the accurate performance of the proposed method. The excellent performance of the proposed method is mainly due to the strong automatic feature learning ability of the proposed CNN model and the generalization ability of the ensemble classifier. 

### 5.2. Case Study 2: Bearing Fault Diagnosis for BaoSteel Rolling Mill

#### 5.2.1. Experimental Setup and Dataset

In this case study, the effectiveness of the proposed method is validated by the bearing condition monitoring data collected from the BaoSteel MRO Management System. For more information, please refer to http://imss.tongji.edu.cn:9080/. The vibration signal data of bearings (here acceleration) are collected from a rolling mill at the frequency of 20 kHz. There are four different health conditions: normal condition (NO), rolling ball defect condition (RD), inner race defect condition (ID), and outer race defect condition (OD). All the samples with 1024 data points are randomly selected from the condition monitoring data. There are 500 training samples and 250 testing samples for each condition.

In this case study, the scale factor of CWT is set as 512. The gray-scale images containing fault information are compressed to the size of 16 × 16 due to the smaller volume of signal data. In addition, the structure of the CNN model is also adjusted and more details are shown in Table 5. It should be noted that the zero padding technique is used to keep the dimension of feature maps unchanged in this case study. The optimal parameters of the CNN model are follows: the batch size is set as 120, the number of epochs is set as 50, and the learning rate is set as 0.05.

#### 5.2.2. Results and Discussion

**Figure 10:**
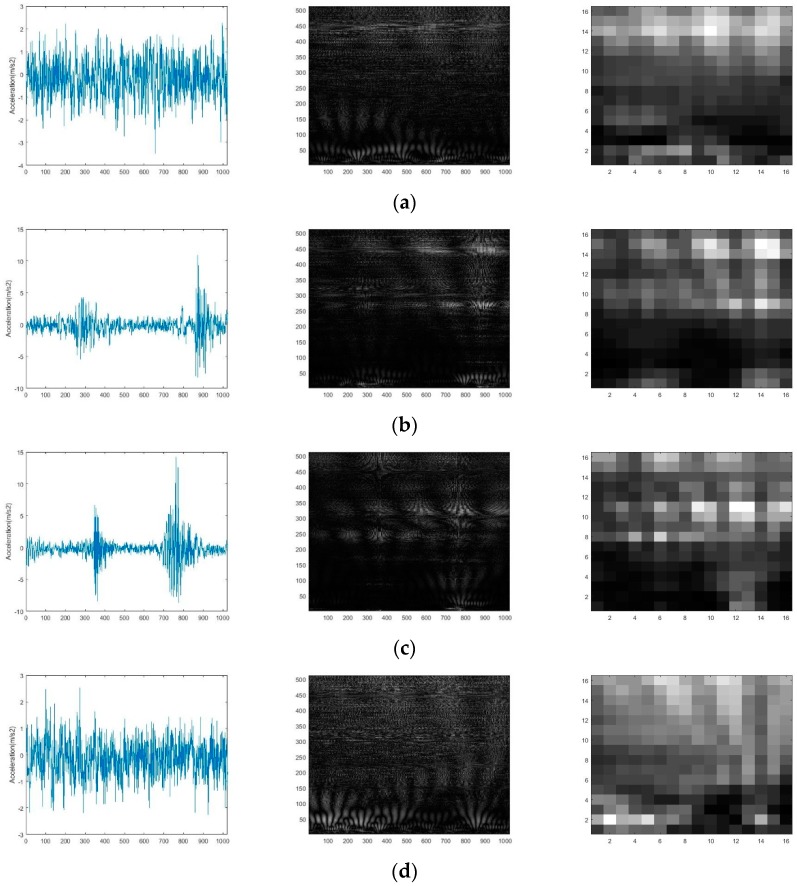
The raw vibration signal waveform and conversion results: (**a**) Normal condition; (**b**) Rolling ball defect condition; (**c**) Inner race defect condition; (**d**) Outer race defect condition.

In this experiment, the same signal-to-image conversion process with case study 1 is executed and the conversion results are shown in Figure 10. 

From the conversion results, it can be seen that the difference between the gray-scale images containing the signal distribution characteristics in the time-frequency domain can be easily detected. This further proves the effectiveness of the conversion method. The CNN model are pre-trained and finally the training accuracy reaches to 99.5% through the weights and bias adjustment. The multi-layer features are also extracted to train different classifiers, the experiments are executed 10 times and the mean and standard deviation of the diagnostic accuracy are shown in Table 6. From Table 6, it can be seen that the method using RF3 classifier based on feature maps in layer FC5 outperforms the method using other classifiers based on feature maps in other layers. Remarkably, the accuracy performance in this case study is weaker than that in the case study 1. Additionally, the architecture of the proposed CNN model is adjusted, but accuracy results exhibit no noticeable improvement. Finally, the raw data are checked. As a result, it was stated that there are some “dirty data” including redundancy data and missing data in the training samples.

#### 5.2.3. Comparison with Other Methods

Table 7 shows the mean accuracies and computation time of the proposed method and traditional methods (BPNN, SVM, DAE, DBN and CNN). It can be clearly seen that the proposed method outperforms all the traditional machine learning based and deep learning based fault diagnosis methods in term of the mean accuracy, showing the great potential of the proposed fault diagnosis method.

## 6. Conclusions and Future Work

In this paper, a novel bearing fault diagnosis method using multi-level features based on CWT, CNN, and RF is proposed. The main contributions of this paper are summarized as follows:

(1) Presenting a novel signal-to-image conversion method based on CWT. The time-frequency spectra obtained from CWT not only fully capture the non-stationary signal characteristics and contain abundant fault information, but also can be regarded as two-dimensional gray-scale images, which are suitable as the input of CNN.
(2) Implementing an automatic feature extractor using CNN. An improved CNN model with optimized configuration is constructed and pre-trained in a supervised manner. The pre-trained model has the ability of extracting sensitive and representative multi-level features automatically from gray-scale images. The extracted multi-level features contain both local and global fault information and can contribute different knowledge to diagnosis results.
(3) Applying the winner-take-all ensemble strategy of multiple RF classifiers to improve the accuracy and generalization performance of the fault diagnosis method. The multi-level features, especially the low-level features in hidden layers, are used for classifying faults independently in this paper. The extracted features in each layer are fed into a RF classifier and each RF classifier separately outputs the diagnostic result. The outputs of all classifiers are combined by the winner-take-all ensemble strategy to generate more accurate diagnostic result.
(4) The proposed method is validated by two bearing datasets from public website and BaoSteel MRO Management System. The former achieves higher diagnostic accuracy at 99.73% than the latter at 97.38%. In particular, the different feature distribution of training and test dataset has almost no effect on the classification accuracy, which indicates the strong generalization ability. The experimental results prove the availability and superiority of the proposed method.

Although the proposed method has made some achievements, there are still two limitations need to be improved in the future work. Firstly, the raw data quality significantly affects the performance of the proposed method and data cleaning process is vital in practical application. Secondly, the enormous training data results in slow convergence speed. The efficiency of the proposed method can be enhanced with the introduction of parallel computing architectures. In addition, the time complexity of the proposed method need to be considered in the design and validation process of deep learning model. For example, there is no need to extract higher-level features of CNN when the low-level features are enough to realize high-precision fault diagnosis, which may contribute to the efficiency improvement. Remarkably, the proposed data-driven method is expected to be widely used in the fault diagnosis of other similar types of rotating machinery, such as gearboxes, pumps, and optical scanning systems.

## Figures and Tables

**Table 1:** The details of the two bearing datasets.

Health Condition Type	Class Label	Dataset I Number of Training (Loads: 0–3)/Testing (Loads: 0–3) Samples	Dataset II Number of Training (Loads: 0–2)/Testing (Loads:3) samples
NO	1	200/200	150/50
IF-0.18	2	200/200	150/50
BF-0.18	3	200/200	150/50
OF-0.18	4	200/200	150/50
IF-0.36	5	200/200	150/50
BF-0.36	6	200/200	150/50
OF-0.36	7	200/200	150/50
IF-0.54	8	200/200	150/50
BF-0.54	9	200/200	150/50
OF-0.54	10	200/200	150/50

**Table 2:** The detailed structure of the CNN model.

Layer Name	Configuration	Kernel/Pooling Size
Input	32 × 32	
C1	6@28 × 28	6@5 × 5
S2	6@14 × 14	2 × 2
C3	12@12 × 12	12@3 × 3
S4	12@6 × 6	2 × 2
FC5	240	
Output	10	

**Table 3:** The mean and standard deviation of accuracy results.

Methods	Accuracy for Dataset I (%)	Standard Deviation (%)	Accuracy for Dataset II (%)	Standard Deviation (%)
CNN + Softmax	99.66	0.101	97.04	0.45
CNN + RF1	99.46	0.243	98.26	0.542
CNN + RF2	99.73	0.109	99.08	0.379
CNN + RF3	99.66	0.128	95.02	0.447
Proposed method	99.73	0.109	99.08	0.379

**Table 4:** The comparison results with other methods.

Methods	Accuracy for Dataset I (%)	Computation Time for Dataset I (s)	Accuracy for Dataset II (%)	Computation Time for Dataset II (s)
BPNN	85.1	18.57	69.8	13.56
SVM	87.45	12.91	74.5	9.12
DBN	89.46	146.94	86.55	109.42
DAE	93.1	139.03	89.4	103.89
CNN	99.66	144.75	97.04	107.58
Proposed method	99.73	152.71	99.08	114.57

**Table 5:** The detailed structure of the CNN model.

Layer Name	Configuration	Kernel/Pooling Size
Input	16 × 16	
C1	32@16 × 16	32@3 × 3
S2	32@8 × 8	2 × 2
C3	64@8 × 8	64@3 × 3
S4	64@4 × 4	2 × 2
FC5	128	
Output	4	

**Table 6:** The mean and standard deviation of accuracy results.

Methods	Accuracy (%)	Standard Deviation (%)
CNN + Softmax	96.67	0.122
CNN + RF1	96.32	0.28
CNN + RF2	96.12	0.193
CNN + RF3	97.38	0.131
Proposed method	97.38	0.131

**Table 7:** The comparison results with other methods.

Methods	Accuracy (%)	Computation Time (s)
BPNN	83.1	20.66
SVM	86.22	14.95
DBN	87.91	151.23
DAE	90.01	142.77
CNN	96.67	150.12
Proposed method	97.38	161.43
